# Computer-Based Training Programs for Older People with Mild Cognitive Impairment and/or Dementia

**DOI:** 10.3389/fnhum.2017.00262

**Published:** 2017-05-16

**Authors:** Blanka Klimova, Petra Maresova

**Affiliations:** ^1^Department of Applied Linguistics, Faculty of Informatics and Management, University of Hradec KraloveHradec Kralove, Czechia; ^2^Department of Economics, Faculty of Informatics and Management, University of Hradec KraloveHradec Kralove, Czechia

**Keywords:** cognitive impairment, dementia, computer programs, efficacy, intervention, older adults

## Abstract

Currently, due to the demographic trends, the number of aging population groups is dramatically rising, especially in developed countries. This trend causes serious economic and social issues, but also an increase of aging disorders such as mild cognitive impairment (MCI) or dementia in older population groups. MCI and dementia are connected with deterioration of cognitive functions. The aim of this mini review article is therefore to explore whether computer-based training programs might be an effective intervention tool for older people with MCI and/or dementia or not. The methods include a literature search in the world’s acknowledged databases: Web of Science, Scopus, Science Direct, MEDLINE and Springer, and consequently, evaluation of the findings of the relevant studies. The findings from the selected studies are quite neutral with respect to the efficacy of the computer assisted intervention programs on the improvement of basic cognitive functions. On the one hand, they suggest that the computer-based training interventions might generate some positive effects on patients with MCI and/or dementia, such as the improvement of learning and short-term memory, as well as behavioral symptoms. On the other hand, these training interventions seem to be short-term, with small sample sizes and their efficacy was proved only in the half of the detected studies. Therefore more longitudinal randomized controlled trials (RCTs) are needed to prove the efficacy of the computer-based training programs among older individuals with MCI and/or dementia.

## Introduction

Mental health affects the quality of life of a large number of individuals and family members (Klimova et al., 2015; Maresova et al., 2016). Dementia is closely linked to aging. Dementia is a consequence of the brain disease and it results in the deterioration of mental and cognitive activities. Most symptoms refers to cognitive impairment, but dementia also affects behavior (Holmerova et al., 2007). Dementia can be diagnosed if there are cognitive and behavioral symptoms which should include at least two of the following aspects (McKhann et al., 2011):

• worsened ability to gain and recall a new source of information (e.g., disorientation in known places, forgetting appointments or asking the same questions several times);

• worsened reasoning and judgment (e.g., low decision-making processes, tendency to safety risks or inability to conduct complex tasks);

• worsened visuospatial skills (e.g., failure to recognize known faces or things, inability to dress appropriately or to find things);

• worsened language (e.g., difficulties finding the right words, name the objects correctly, making hesitations or writing mistakes); and

• behavioral changes (e.g., frequent changes in mood, social withdrawal or apathy).

Mild cognitive impairment (MCI) is then one of the pre-phases in the development of Alzheimer’s disease (AD) and people usually have moderate difficulties with cognitive functions such as memory, attention, language or vision.

Although there already exist a few effective drugs to delay the cognitive decline and the development of dementia (Lyketsos et al., 2011; Klimova and Kuca, 2016), these drug therapies still have, however, quite modest benefits and they are rather expensive (Maresova et al., 2016). Therefore there is ongoing effort to use non-pharmacological approaches as a good support in the treatment of dementia (Klimova and Kuca, 2015; Klimova et al., 2016). One of such support, with the emergence and abundant use of information and communication technologies, seems to be computer-based intervention programs which may contribute to the maintenance and in some cases, to the reduction of cognitive disorders in dementia, particularly in the early stages of this disease as the research studies confirm (Preece and Maloney-Krichmar, 2003; Savitch et al., 2006). These computer programs can be tailored-made and meet patients’ needs. They are also economical and can be more easily disseminated among a wide range of people (Klimova and Maresova, 2016). Recent research studies (Hernandez-Encuentra et al., 2009; Sayago et al., 2011; Kueider et al., 2012) have indicated that older generation groups are now more technologically savvy than two decades ago. However, most of the computer-based training programs are aimed at healthy older individuals such as three well-known commercial, computer-based training programs CogMed, Jungle Memory and Cognifit (Fernandez, 2011; Melby-Lervåg and Hulme, 2013). Thus, more research has been conducted among healthy older individuals and it has shown some evidence of training with the help of modern technologies on cognitive performance as several reviews (see Kueider et al., 2012; Klimova, 2016) and meta-analytic studies have illustrated (see Powers et al., 2013; Lampit et al., 2014; Toril et al., 2014).

The aim of this mini review article is therefore to explore whether computer-based training programs might be an effective intervention tool for older people with MCI and/or dementia or not.

## Methods

The methodology of this mini review article follows Moher et al. (2009). The methods involved in this study include a review of available literature sources dealing with the research topic. Furthermore, a method of comparison of findings of different research studies exploring the research topic was applied. Research studies were selected on the basis of the following keywords:

• *cognitive impairment* AND *computer-based training programs*,

• *dementia* AND *computer-based training programs*,

• *computer-based training programs* AND *Alzheimers disease*.

These keywords were found in research studies in peer-review English written articles from the Web of Science, Science Direct, Springer, Scopus, and MEDLINE in the period of 2000 up to the end of 2016. However, there were not many studies on the efficacy of computer-based training programs on people with MCI and/or dementia. The lack of the studies dealing with computer-based training programs has been also confirmed in literature (Barnes et al., 2009). Most of the studies were found in the database Science Direct, almost 4976, in Scopus—506 documents, in Web of Science—altogether 196 studies and 210 studies in Medline. The studies were first screened for the title of their abstracts and duplications. Then, the abstracts were screened and eventually, the full-text. After this, only six studies were detected for the final review although there are some studies in progress, such as Flak et al. (2014). The study was included if it matched the corresponding period, i.e., from 2000 to the end of 2016, involved older people with MCI and/or dementia, focused on computer assisted training, respectively computer assisted cognitive training, was a randomized controlled trial (RCT), and was written in English. The search started with the year of 2000 because this is the period when the studies on computer-based training programs among older individuals began to appear. Furthermore, besides dementia, the authors of this mini review article also included the studies on MCI since MCI represents a transitional stage between aging and AD. The high rate of transition from MCI to AD makes early treatment an important clinical issue (see Rozzini et al., 2007). As Lu et al. (2011) claim, RCT are regarded to be the best design plan to verify the efficacy of intervention measures because they require rigorous design and scientific methods.

The selection process of the studies was as follows:

1. The search was conducted according to the keywords in four databases (5888) and in other web sources (32).
2. Furthermore, duplication was removed. Altogether 3718 studies were identified. Afterwards, the titles of only 276 studies/articles were checked in compliance with the research purpose within the Web of Science, Scopus a Medline, which contain the studies on clinical trials. In fact, this type of articles was included in the inclusion criteria. Thus, altogether 276 studies were selected for the further analysis.
3. The authors checked the content of the abstracts in order to discover whether the study examines the research topic. After that, 98 studies/article were selected for the full-text analysis.
4. Altogether 42 studies focused on the research topic, however, only six studies could be eventually used for a detailed specification. These were publications whose findings were new and contained traceable and comparable data such as a number of subjects or results specifications.

## Findings

Altogether six studies were identified for this review article. All studies were RCTs. Thus, the subjects were randomly divided into an experimental and control group. In all studies the control groups were active, exposed to traditional cognitive training (TCT) using pen-and-paper exercises designed to improve cognitive functions: attention and concentration, memory, language, calculation, or orientation to reality, or the subjects in the control group were involved in an integrated psychostimulation programs (IPPs) comprising music therapy, art and crafts and physical activity. Only in one trial (Tárraga et al., 2006) there were two control groups; one active and one passive. The mean age of subjects ranged between 74 years and 83 years. The dose of the intervention period was between 4 weeks and 24 weeks. The efficacy of computer-based intervention programs on cognitive impairment among older individuals with MCI and dementia was measured by validated neuropsychological tests such as digit span test, vocabulary recall test, or verbal fluency test. Three studies (Tárraga et al., 2006; Barnes et al., 2009; Herrera et al., 2012) confirmed beneficial effects of computer-based intervention programs on cognitive functions such as episodic memory or abstract reasoning, while the other three (Galante et al., 2007; Gaitan et al., 2012; Yu et al., 2015) revealed no effect of this intervention on cognitive functioning among older people with MCI and/or dementia. The description of the analyzed studies and their findings are summarized in alphabetical order of their first author in Table 1 below.

**Table 1:** **An overview of the selected studies on computer-based intervention programs for cognitively impaired people with mild cognitive impairment (MCI) and/or dementia and their efficacy**.

Study	Objective	No. of subjects, age range	Dose of interventions, follow-up period, active vs. passive control group	Main outcome measures	Experimental group intervention	Control group intervention	Findings, statistically significant differences
Barnes et al. (2009) RCT	To determine whether intensive computer-based cognitive training is feasible in subjects with MCI and to estimate the size of its effect on cognition.	47 subjects with MCI, mean age: 74 years; age range: 54–91 years.	100 min/day, 5 days/week for 6 weeks; no follow-up period; active control group.	Repeatable battery for assessment of neuropsychological status.	22 subjects did exercises specifically designed to improve auditory processing speed and accuracy.	The control group subjects (*N* = 25) performed more passive computer activities (reading, listening, visuospatial game).	The findings indicate that intensive, computer-based mental activity is feasible in subjects with MCI and that these training programs may have domain specific effects because the intervention group improved in verbal learning and memory measures (range, 0.16–0.53), while the control group in language and visuospatial function measures (range, −0.51 to 0.01).
Gaitan et al. (2012) RCT	To evaluate the efficacy at 12 months of a computer-based cognitive training (CBCT) program, adjunctive to traditional cognitive training (TCT).	60 patients with multi-domain mild cognitive impairment and mild Alzheimer’s disease; mean age: 75.82 years; age range: 57–85 years.	30 sessions of 1 h, two or three times a week for 12 weeks; 12-month follow-up; active control group.	Battery of neuropsychological tests.	37 subjects participated in CBCT and TCT.	The control group subjects (*N* = 23) only performed TCT.	The results reveal that The addition of a CBCT program was effective in anxiety (*p* = 0.03) and decision making (*p* = 0.04) but had no significant effects on outcomes in basic cognitive functions.
Galante et al. (2007) RCT	To explore the efficacy of computer cognitive rehabilitation in patients with mild cognitive decline.	11 subjects with AD and MCI; mean age: 76 (± 6.0), years; age range: not stated.	12 individual 60 min sessions of training, three times per week for 4 weeks; in addition, there was a 3-month and a 9-month follow-up period; active control group.	Neuropsychological tests.	Seven subjects were doing specific treatment—computer exercises focused on cognitive functions.	The control group subjects (*N* = 4) attended the aspecific treatment—a semi-structured interview on current affairs and relevant events of their own life history.	The Mini Mental State Examination (MMSE) score of the control group decreased significantly at the 9-month follow-up with respect both to baseline (*p* = 0.04) and to the 3-months follow-up (*p* = 0.008), while the mean MMSE score of the experimental remained stable over time. Generally, the findings show that computer training technique is effective, at least in delaying the continuous progression of cognitive impairment in AD.
Herrera et al. (2012) RCT	To evaluate the efficacy of a 12-week computer-based memory-attention training program based on recognition in subjects with MCI.	22 subjects with amnestic MCI multiple domains subtype; mean age: 75 years; age range: 65–90 years.	24 sessions of 1 h for 12 weeks; 6 month follow-up; active control group.	Neuropsychological tests, i.e., digit-span test, the 12-word-list recall test from the BEM-144 memory battery and cued reminding test.	The experimental group (*N* = 11) was doing a computer-based memory-attention training program.	11 subjects of the control group were trained in cognitively stimulating activities.	The findings show that the intervention group improved episodic recall and recognition in comparison with the control group although only recognition was trained.
Tárraga et al. (2006) RCT	To determine the usefulness of an interactive multimedia internet-based system (IMIS) for the cognitive stimulation of Alzheimer’s disease.	43 mildly impaired patients, suspected of having Alzheimer’s disease; mean age: 77 years; age range: not stated.	12 weeks of treatment; a 24-week follow-up period; one active and one passive control group.	Alzheimer’s Disease Assessment Scale-Cognitive, Mini-Mental State Examination, Syndrom Kurztest, Boston Naming Test, Verbal Fluency, and the Rivermead Behavioral Memory Test story recall subtest.	15 subjects of the experimental group had 3 weekly, 20-min sessions of IMIS in addition to 8 h/day of an integrated psychostimulation program (IPP) and cholinesterase inhibitors (ChEIs) treatment.	One IPP and ChEIs treatment control group (*N* = 16); and one control group (*N* = 12) with only ChEIs treatment.	After 12 weeks, the patients treated with both IMIS and IPP had improved outcome scores on the Alzheimer’s Disease Assessment Scale-Cognitive (ADAS-Cog) and MMSE, which was maintained through 24 weeks of follow-up. Both the IPP and IMIS improved cognition in patients with Alzheimer’s disease, the IMIS program provided an improvement above and beyond that seen with IPP alone, which lasted for 24 weeks.
Yu et al. (2015) RCT	To explore the potential benefits of a computer-assisted intervention using touch-screen videogame technology on cognitive function and behavioral symptoms in older adults with mild-to-moderate dementia.	32 subjects with mild-to-moderate dementia; mean age: 83 years; age range: 70–99 years.	30 min per session, 1–2 sessions per week for a total of eight sessions; 4 weeks of follow-up; active control group.	Montreal Cognitive Assessment, digit span, the category verbal fluency tests, Neuropsychiatric Inventory test.	The intervention group subjects (*N* = 16) had a videogame training.	The control group (*N* = 16) performed a traditional cognitive training.	The experimental group showed significant improvements in MoCA language sub-scores (pre 1.5, post 2.0, *P* < 0.05, Effect Size (ES) 0.82). In addition, the findings indicate that the touch-screen videogame training can alleviate behavioral symptoms in older adults with mild-to-moderate dementia. Its efficacy to improve cognitive and other related functions warrants further investigation.

*Explanation: AD, Alzheimer’s disease; aMCI, Amnestic Mild Cognitive Impairment; IMIS, interactive multimedia internet-based system; IPP, integrated psychostimulation program; MCI, Mild Cognitive Impairment; MSA, multiple system atrophy; RCT, randomized controlled trial*.

## Discussion of the Findings

As Table 1 above demonstrates, the findings of this mini review article are quite modest and neutral on the issue of the efficacy of computer-based training interventions on cognitive decline among older people with MCI and/or dementia because half of the presented studies suggest small benefits for the improvement of cognitive performance among older individuals with MCI and/or dementia and the other half of the studies do not show any effects on cognitive decline.

As the results of the detected trials indicate, the positive effects on the improvement of cognitive performance among older adults with MCI and/or dementia concern especially the following areas: verbal and visuospatial memory, episodic memory, verbal learning, attention and decision-making. For example, Barnes et al. (2009) reported improvements of verbal learning and memory and observed an effect Size (ES) of 0.33 SD as far as the global cognitive function is concerned. Herrera et al. (2012) on the *post hoc* Newman–Keuls *t*-test (*p* < 0.05) showed that the trained group had reached the better results in recognition and recall of words than the control group. The same was true for the study by Tárraga et al. (2006). Similar findings were also described by Coyle et al. (2015) who in their review state that especially the cognitive domains of visual and verbal memory and executive functions could be improved. This has been also confirmed by Cipriani et al. (2006).

Although the rest of the studies did not prove any effect on cognitive decline among the target group (Galante et al., 2007; Gaitan et al., 2012; Yu et al., 2015), their results showed that computer-based training may be effective at least in delaying the continuous progression of cognitive impairment in AD (Galante et al., 2007). Furthermore, Gowans et al. (2007) in their study conducted among 40 people with AD and their 30 caregivers indicate that even in later stages of AD people can interact meaningfully when prompted specifically. The computer-based training may help them with reminiscence, communication as well as social contact. In addition, the findings of this mini review article confirm a positive effect of the computer-based intervention on behavioral symptoms such as depression and anxiety on older people with MCI and/or dementia (Yu et al., 2015). A more recent review study by Garcia-Casal et al. (2016) also argue that computer-based cognitive interventions have moderate effects on cognition, depression and anxiety in people with dementia and no significant effects can be found on activities of daily living.

The findings of the detected randomized control trials generated several important issues since they differed on numerous dimensions that might have affected the results of the training. First, the age range of the subjects was quite wide, for instance 54–91 years (Barnes et al., 2009) or 57–85 years (Gaitan et al., 2012) and the researchers did not study the effects of younger-older adults or older-older adults as, for instance, was done in the meta-analytic studies by Powers et al. (2013), Lampit et al. (2014) and Toril et al. (2014) who performed research on this issue among healthy older individuals. They revealed that there had been small benefits after training with larger effects in old-older adults than in young-older adults. However, in a recent meta-analysis by Wang et al. (2016), young adults have benefited more from the cognitive training than the older adults. Second, there was a question of the duration of these trainings. Some research studies suggest that that longer and more intense interventions might contribute to the improvement of cognitive functions among people with MCI and/or dementia (Bozoki et al., 2013). On the contrary, Ballesteros et al. (2014) or Toril et al. (2014) claim that the effects of cognitive training in healthy old individuals are greater when training is of short duration (1–6 weeks) than when it is long (7–12 weeks). Third, the study designs of the selected studies also differed in the type of intervention programs. Only two studies, for example, had the same type of training for their control groups.

Thus, the limitations of this review article were quite significant since the identified studies consisted of the small sample sizes, large variability of study designs and outcome measures used in the analyzed RCTs, which may have had ambiguous results due to the overestimated effects of training interventions. Moreover, apart from the study by Garcia-Casal et al. (2016), the authors did not manage to detect any negative studies on this issue, which contributes to publication bias since studies with negative results tend to be less likely published. For these reasons and the reasons described above, the authors of this study could not conduct the meta-analysis.

## Conclusion

The findings from the selected studies seem to be quite neutral with respect to the efficacy of the computer assisted intervention programs on the improvement of basic cognitive functions. On the one hand, they suggest that the computer-based training interventions might generate some positive effects on patients with MCI and/or dementia, such as the improvement of learning and short-term memory, as well as behavioral symptoms. On the other hand, these training interventions seem to be short-term, with small sample sizes, and their efficacy, as far as the statistically significant differences are concerned, was proved only in the half of the detected studies. Therefore there is a need for more longitudinal RCTs which would prove the efficacy of these computer-based training programs as a suitable intervention tool for cognitively impaired older people since the pharmacological treatment generates quite modest benefits, it is invasive, and rather expensive. Furthermore, earlier diagnosis of cognitive degeneration, with the accurate assessment tools (see Vestal et al., 2006) may help not only to assign the right medication treatment but also the management of cognitive disorders.

## Author Contributions

BK and PM equally contributed to the drafting, analyses and final version of the whole manuscript. Both authors read and approved the final manuscript.

## Conflict of Interest Statement

The authors declare that the research was conducted in the absence of any commercial or financial relationships that could be construed as a potential conflict of interest.
